# Impact of Loop Electrosurgical Excision (LEEP/LLETZ) on the Quality of Sexual Life in Women of Reproductive Age—A Prospective Longitudinal Study

**DOI:** 10.3390/jcm14082787

**Published:** 2025-04-17

**Authors:** Barbara Suchońska, Michalina Sikorska, Agata Majewska, Monika Dominiak, Daria Salloum, Anna Antosik-Wójcińska, Paweł Mierzejewski, Aleksandra Zyguła

**Affiliations:** 1 1st Department of Obstetrics and Gynecology, Medical University of Warsaw, 02-015 Warsaw, Poland; 2 Department of Pharmacology, Institute of Psychiatry and Neurology, 02-957 Warsaw, Poland; sikorskamichalina@gmail.com (M.S.); mdominiak@ipin.edu.pl (M.D.); mierzeje@ipin.edu.pl (P.M.); 3 Department of Medicine, National and Kapodistrian University of Athens, 11527 Athens, Greece; 4 Department of Obstetrics and Gynecology, Institute of Mother and Child, 01-211 Warszawa, Poland; majewska.agata@gmail.com; 5 Doctoral School, Medical University of Warsaw, 02-091 Warsaw, Poland; daria.salloum@wum.edu.pl; 6 Department of Psychiatry, Faculty of Medicine, Collegium Medicum, Cardinal Wyszyński University, 01-938 Warsaw, Poland; antosikwojcinska@gmail.com; 7 OVIklinika, 01-377 Warsaw, Poland; am.zygula@gmail.com

**Keywords:** loop electrosurgical excision procedure, LEEP/LLETZ, human papillomavirus, HPV, psychosocial distress, cervical cancer, sexual dysfunction

## Abstract

**Background**: Cervical cancer is one of the most common cancers in women worldwide, with the leading risk factor being high-risk human papillomavirus (HR-HPV); persistent HR-HPV infection leads to cervical dysplasia. With early screening and, if indicated, therapeutic strategies such as a loop electrosurgical excision procedure (LEEP) and large loop excision of the transformation zone (LLETZ), morbidity and mortality in this population are decreasing. However, it is suspected that these procedures may have an impact on sexual dysfunction. **Methods**: In this single-center prospective longitudinal study, we recruited patients with a high-grade squamous intraepithelial lesion (HSIL) and HR-HPV-positive result and evaluated the impact of LEEP/LLETZ on their sexual life and psychological well-being. All participants received two questionnaires—the Female Sexual Function Index (FSFI) and the Brief Index of Sexual Function-Women (BISF-W)—after diagnosis, before treatment, and three months after the procedure. **Results**: A total of 40 women aged 28 to 55 years were enrolled. This study showed no significant changes in both the FSFI (F(1,39) = 0.774; *p* = 0.38) and BISF-W total scores (F(1,39). This study revealed that 32/40 (80%) of participants based on the FSFI either exhibited no change or improved sexual function. Only 3/40 (7.5%) mentioned sexual dysfunction after procedures. This study also found that the mean score for sexual function based on the FSFI was 2.80; *p* = 0.102. **Conclusions**: These findings suggest that patients who qualified for LEEP/LLETZ can be reassured that the anxiety they experience prior to treatment is not necessarily justified. This provides evidence of the safety of loop excision procedures in terms of sexual functioning after the procedure. Nevertheless, further studies are needed to analyze the potential risk factors that may contribute to adverse sexual outcomes and to achieve a better understanding of this complex problem.

## 1. Introduction

Cervical cancer (CC) is the fourth most common cancer in women worldwide. The advent of cervical cancer screening has led to the early detection of precancerous squamous intraepithelial lesions (SIL), resulting in a decline in morbidity and mortality among patients. Various risk factors for CC have been identified, including high-risk human papillomavirus (HR-HPV) infection, with HPV 16 and 18 being the predominant types contributing to SIL. It is established that persistent HR-HPV infection can lead to high-grade squamous intraepithelial lesions (HSIL CIN2/3) and, consequently, progression to invasive CC [1,2,3,4]. Cervical cancer screening encompasses HPV genotyping and cytology. In the event of an HR-HPV-positive result and abnormal cytology, women are referred to colposcopy in accordance with a standardized protocol [5]. If a lesion is detected during colposcopy, a guided biopsy is performed to establish a histopathological diagnosis. In cases of HSIL CIN2/3, patients are eligible for one of the following excision procedures of the pathological cervix: the loop electrosurgical excision procedure (LEEP) or large loop excision of the transformation zone (LLETZ). The excisional techniques employed in such cases are office-based, making use of an electrosurgical loop for cervical conization with an excision depth of 7–25 mm. Nevertheless, patients following these procedures may experience concerns regarding their sexual functioning, as LEEP/LLETZ has the potential to result in impairment to the vascular supply and innervation of the cervix, which can subsequently lead to dyspareunia and sexual dysfunction [6]. Furthermore, the diagnosis of HSIL CIN2/3 can induce anxiety, which can, in turn, affect the quality of sexual intercourse. Previous studies have demonstrated that anxiety and depressive symptoms can persist for months following excision procedures [7,8,9]. The extant literature offers equivocal results with regard to the impact of LEEP/LLETZ procedures on sexual functioning [8,9,10,11,12,13,14]. Two studies demonstrated that patients post-procedure reported diminished sexual satisfaction [8,10]. Furthermore, Giovannetti et al. found that these women exhibited altered sensory perception (numbness), dyspareunia, and decreased libido [11]. Conversely, three other studies found no significant difference in sexual functioning between patients after the excision procedure and the control group [9,13,14]. Intriguingly, one study noted an improvement in dyspareunia and postcoital bleeding after LEEP treatment [12]. A decision was taken to perform a prospective cohort study on HSIL, CIN2/3, and qualified LEEP/LLETZ due to the paucity of studies on the subject, the inconsistency of the data available, and the relevance of proper sexual functioning. The aim of this study was to investigate the impact of excision procedures on the sexual life and psychological well-being of the population concerned.

## 2. Materials and Methods

The present study was conducted using a prospective longitudinal approach at the 1st Department of Obstetrics and Gynaecology, Medical University of Warsaw, Poland, between October 2017 and August 2018. The inclusion criteria comprised the following: (1) age above 18 years old, (2) HR-HPV-positive result, (3) cytological high-grade squamous intraepithelial lesions (HSIL) result, (4) fluency in Polish, and (5) no history of an excision procedure. The exclusion criteria comprised the following: (1) pregnancy and (2) other neoplastic diseases. This study was approved by the Ethics Committee at the Medical University of Warsaw, Poland. Patients who met the inclusion criteria were given two questionnaires: the Female Sexual Function Index (FSFI) and the Brief Index of Sexual Functioning for Women (BISF-W). These were given to patients three months prior to and three months following LEEP/LLETZ.

### 2.1. FSFI

The Female Sexual Function Index (FSFI) is a 19-item questionnaire designed to assess female sexual function and sexual arousal disorders (FSAD). It comprises six domains of sexual function: sexual desire (questions 1 and 2), arousal (questions 3–6), lubrication (questions 7–9), orgasm (questions 10–13), satisfaction (questions 14–16), and pain (questions 17–19) (see Supplementary Materials) [15]. The FSFI provides a precise and comprehensive evaluation of each phase of female arousal and pleasure, aligned with the Masters and Johnson sexual response curve: arousal, plateau, orgasm, and resolution. Scores range from 2 to 36, with patients receiving 1–5 points for questions 1, 2, 15, and 16, and 0–5 points for items 3–14 and 17–19 [16,17]. Higher scores indicate better sexual function.

### 2.2. BISF-W

The Brief Index of Sexual Functioning for Women (BISF-W) questionnaire is a 22-item instrument designed to evaluate the current level of female sexual function. It is grouped into seven domains: D1 (desire), D2 (arousal), D3 (frequency of sexual activity), D4 (receptivity/initiation), D5 (pleasure/orgasm), D6 (relationship satisfaction), and D7 (problems affecting sexual satisfaction) (see Supplementary Materials). Scores on the BISF-W range from 16 to 75, with higher scores indicating better sexual function [18]. The impact of the LEEP procedure on sexual function was assessed using the FSFI as the primary scale and the BISF-W as the secondary scale. The complex correlation between medical interventions, psychological factors, and sexual health in the context of HR-HPV-positive results and cervical dysplasia was analyzed using the questionnaires. The primary outcome was the mean difference in the FSFI total score before and after the excision procedure. The mean changes in domain scores in both the FSFI and BISF-W questionnaires after LEEP/LLETZ were also reported. Secondary outcomes were based on the FSFI cut-off point of 26.55, with a value below this indicating the presence of sexual functioning problems [19]. The percentage of women who exhibited a change in sexual functioning, i.e., the cessation of sexual intercourse after the excision procedure, was calculated based on the cut-off point on the FSFI scale. To the best of our knowledge, this is the first study to evaluate the impact of LEEP/LLETZ on the quality of sexual life in reproductive-aged women using validated tools, i.e., the FSFI and BISF-W questionnaires.

### 2.3. Statistical Analysis

Continuous variables were described as the mean ± standard deviation (SD) or medians with an interquartile range (IQR). The Shapiro–Wilk test was employed to evaluate the distribution of the data.

To facilitate a comparison of the FSFI and BISW-W scores before and after the excision procedure, a repeated measures analysis of variance (ANOVA) was employed. Statistically significant results were defined as *p*-values less than 0.05. Statistical analysis was performed by the TIBCO Statistica 13.3 (version 13.3) software.

## 3. Results

A total of 40 eligible women were recruited at the 1st Department of Obstetrics and Gynaecology, Medical University of Warsaw (Poland), between October 2017 and August 2018. Precisely 6/40 (15%) women had no sexual activity four weeks prior to the questionnaire before the excision procedure, whereas 3/40 (7.5%) declared no sexual intercourse four weeks prior to the second questionnaire after the treatment.

### 3.1. Primary Outcome

Table 1 presents the FSFI domain scores before and after the excision procedure. The investigation revealed no statistically significant variation in the overall FSFI total score before and after LEEP/LLETZ (F(1,39) = 0.774; *p* = 0.381). Furthermore, no significant changes in the mean scores in any of the FSFI domains were observed before and after the procedure.

### 3.2. Secondary Outcomes

**Figure 1:**
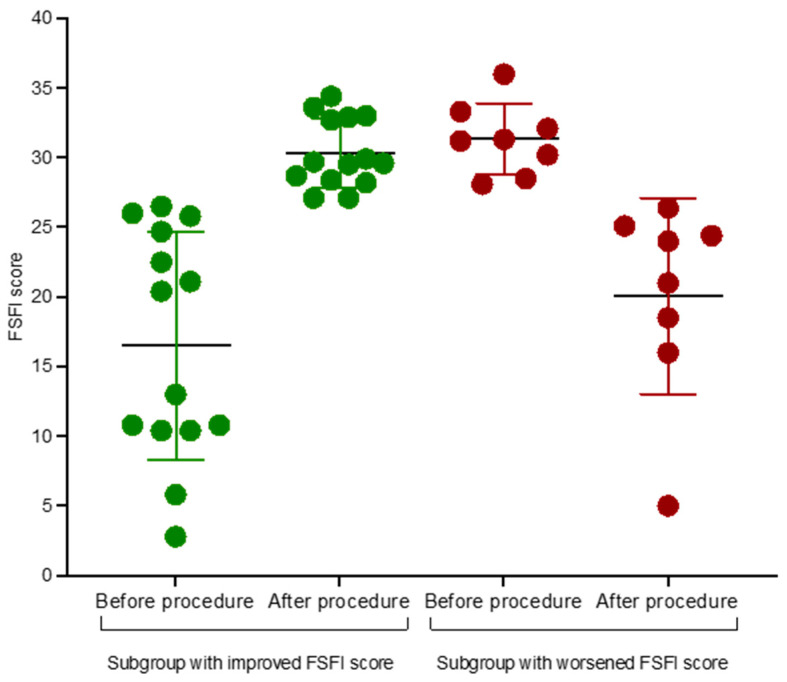
Status change in women prior to and after LEEP/LLETZ (based on the cut-off point on the FSFI scale). Data presented as individual values, means, and standard deviation.

The investigation revealed that 18/40 (45%) of the subjects experienced sexual dysfunction, whereas 22/40 (55%) exhibited normal sexual functioning prior to the excision procedure, as determined by the cut-off point in the FSFI. Following LEEP/LLETZ, 22/40 (55%) patients exhibited a change in status, with 14/22 (63.6%) women demonstrating an improvement in their score and 8/22 (36.3%) experiencing a deterioration (Figure 1).

In total, 32/40 (80%) cases exhibited either stability or enhancement in sexual functioning following the excision procedure. In addition, the BISF-W did not reveal any significant difference in the total score before and after the excision procedure (F(1,39) = 2.80; *p* = 0.102). Furthermore, no significant changes in domain scores before and after LEEP/LLETZ were found (Table 2).

## 4. Discussion

Human papillomavirus (HPV) infection is one of the most prevalent sexually transmitted infections worldwide. Persistent infection has been demonstrated to heighten the risk of HSIL, CIN2/3, and excision procedures. Previous studies have indicated that LEEP/LLETZ can result in anxiety regarding postprocedural sexual functioning [20]. While numerous questionnaires have been described for the evaluation of female sexual function, only a limited number meet the requisite scientific quality standards, including the FSFI and the BISF-W [15,18]. The present study provides novel data on sexual functioning and psychological well-being in patients who underwent LEEP/LLETZ. Conversely, previous studies have yielded conflicting results, with some indicating improvement and others reporting sexual dysfunction among women post-excision procedures [8,10,11,12,21]. A limitation of previous studies was the use of a single questionnaire, which focused exclusively on patients’ sexual lives [13,14]. In contrast, our study utilized two standardized tools, the FSFI and BISF-W, and found no significant changes in overall satisfaction in sexual life after LEEP/LLETZ. This observation pertains to both the aggregate scores and the individual domains of the aforementioned questionnaires. These findings provide evidence to suggest that loop excision procedures do not have an adverse effect on sexual functioning. It has been indicated that patients with an HPV (human papillomavirus)-positive result who have undergone an excision procedure have higher levels of anxiety [8,22,23,24,25,26]. However, the present study contradicts this hypothesis by demonstrating that concerns regarding sexual function subsequent to LEEP/LLETZ are unwarranted, as no substantial impairment in sexual activity was observed following the procedure. Of particular interest is the finding that the domains pertaining to desire and arousal exhibited the lowest scores. These domains may be associated with the psychological state and potential fear associated with a positive HPV result or LEEP/LLETZ procedure. As desire and arousal are directly linked to a woman’s mental health, a lack of professional psychological and sexological care may have an impact on achieving arousal anxiety and even lead to depression in these women [27,28,29,30,31,32,33,34,35,36]. Consequently, healthcare professionals require comprehensive training to recognize the necessity for psychological support and appropriate education in such cases. This study demonstrates that excision procedures have no detrimental effect on sexual function. It is a pioneering effort to dispel uncertainties by utilizing two sexual functioning questionnaires. Nevertheless, this study acknowledges the need for further research in the field of sexual education, particularly with regard to the dissemination of information regarding sexually transmitted diseases. The provision of comprehensive and proficient insights regarding medical conditions or proposed procedures to patients assumes paramount significance, promising a substantial reduction in psychological distress and anxiety. Furthermore, it is acknowledged that every woman’s experience with HPV infection and excision procedures may vary. Consequently, psychological and sexological care should be tailored to the distinctive requisites and inclinations of each patient, ensuring the most favorable outcomes. Nonetheless, the findings of this study indicate that eight participants had FSFI scores below the cut-off point for sexual dysfunction, and in three cases, both questionnaires indicated a deterioration in sexual functioning after loop excision procedures.

The impact of demographic and clinical variables on these outcomes cannot be discounted; however, the study’s limited sample size precludes such an analysis. Consequently, the analysis of this variable in future research would be a beneficial addition.

## 5. Conclusions

This study demonstrates that LEEP/LLETZ does not have a significant effect on sexual functioning. Moreover, anxiety associated with these procedures in the context of subsequent sexual activity appears to be unwarranted. However, the results of this study call for the implementation of educational initiatives aimed at enhancing public awareness regarding loop excision procedures. It is recommended that further studies be conducted with a larger cohort of women following excision procedures, with the analysis adjusted for other clinical and demographic factors, to enhance understanding of this complex problem.

## Figures and Tables

**Table 1:** The Female Sexual Function Index (FSFI): total and domain scores in women prior to and following LEEP/LLETZ procedure. SD—standard deviation.

Dimension	Before Excision Procedure (n = 40)	After Excision Procedure (n = 40)	F	*p*-Value
Desire (mean ± SD) Median	3.8 ± 1.3 3.6	3.8 ± 1.2 4.2	F(1,39) = 0.003	*p* = 0.842
Arousal (mean ± SD) Median	4.0 ± 1.9 4.5	4.3 ± 1.5 4.7	F(1,39) = 0.611	*p* = 0.439
Lubrication (mean ± SD) Median	4.2 ± 2.1 4.9	4.7 ± 1.6 5.1	F(1,39) = 1.174	*p* = 0.285
Orgasm (mean ± SD) Median	4.0 ± 2.1 4.8	4.3 ± 1.7 4.8	F(1,39) = 0.509	*p* = 0.479
Satisfaction (mean ± SD) Median	4.7 ± 1.2 5	4.7 ± 1.4 5.2	F(1,39) = 0.004	*p* = 0.948
Dyspareunia (mean ± SD) Median	4.5 ± 1.5 4.8	5.1 ± 1.4 5.6	F(1,39) = 2.459	*p* = 0.124
Total (mean ± SD) Median	25.1 ± 8.8 28.0	26.8 ± 7.5 28.8	F(1,39) = 0.774	*p* = 0.381

**Table 2:** The brief index of sexual functioning for women (BISF-W): total and domain scores in women prior to and following LEEP/LLETZ procedure. SD—standard deviation.

Dimension	Before Excision Procedure (n = 40)	After Excision Procedure (n = 40)	F	*p*-Value
D1 Thoughts/Desire (mean ± SD) Median	4.75 ± 2.7 4.14	5.31 ± 3.04 5.57	F(1,39) = 0.936	*p* = 0.339
D 2 Arousal (mean ± SD) Median	6.97 ± 2.81 7.5	7.3 ± 3.13 8.25	F(1,39) = 0.253	*p* = 0.617
D3 Frequency of sexual activity (mean ± SD) Median	4.18 ± 2.67 3.88	4.43 ± 2.82 4	F(1,39) = 0.163	*p* = 0.688
D4 Receptivity/initiation (mean ± SD) Median	7.15 ± 4.11 8	7.9 ± 2.72 8	F(1,39) = 0.918	*p* = 0.343
D5 Pleasure/orgasm (mean ± SD) Median	4.83 ± 3.07 5.13	5.97 ± 2.9 6.5	F(1,39) = 2.827	*p* = 0.100
D6 Relationship satisfaction (mean ± SD) Median	8.55 ± 3.09 9	9.05 ± 2.96 10	F(1,39) = 0.627	*p* = 0.433
D7 Problems affecting sexual functioning (mean ± SD) Median	4.76 ± 2.63 4.71	4.54 ± 1.85 4.61	F(1,39) = 0.192	*p* = 0.663
Composite score (mean ± SD) Median	31.12 ± 13.89 31.75	35.62 ± 11.33 36.87	F(1,39) = 2.804	*p* = 0.102

## Data Availability

The questionnaires completed by the patients were archived at the 1st Department of Obstetrics and Gynecology, Medical University of Warsaw. The files to which the analyzed data were transferred are available to the authors and can be made available to interested parties but have not been publicly archived. Despite being anonymized, the files contain intimate and sensitive data.

## Supplementary Materials

The following supporting information can be downloaded at: https://www.mdpi.com/article/10.3390/jcm14082787/s1, FSFI fsfi_test.pdf, BISF-W in ref. [13].

## Abbreviations

The following abbreviations are used in this manuscript:

HSIL	High-grade squamous intraepithelial lesions
CC	Cervical cancer
HR HPV	High-risk human papillomavirus
HPV	Human papillomavirus
LEEP	Loop electrosurgical excision procedure
LLETZ	Large loop excision of the transformation zone
FSFI	Female sexual function index
BISF-W	Brief index of sexual function-women
FSAD	Function and sexual arousal disorders
IQR	Interquartile range
