# Supplementary material for: Impact of Loop Electrosurgical Excision (LEEP/LLETZ) on the Quality of Sexual Life in Women of Reproductive Age—A Prospective Longitudinal Study
Source: J Clin Med. 2025 Apr 17;14(8):2787. doi: 10.3390/jcm14082787 (PMC12028190; doi:10.3390/jcm14082787)
Supplement: Supplementary file 1 [file jcm-14-02787-s001.zip › jcm-3507611-supplementary.pdf]

Female Sexual Function Index
(FSFI) *

**Question**	**Response Options**
**Q1**: Over the past 4 weeks, how **often** did you feel sexual desire or interest?	◯ 1 = Almost always or always<br>◯ 2 = Most times (more than half the time)<br>◯ 3 = Sometimes (about half the time)<br>◯ 4 = A few times (less than half the time)<br>◯ 5 = Almost never or never
**Q2**: Over the past 4 weeks, how would you rate your **level** (degree) of sexual desire or interest?	◯ 1 = Very high<br>◯ 2 = High<br>◯ 3 = Moderate<br>◯ 4 = Low<br>◯ 5 = Very low or none at all
**Q3**: Over the past 4 weeks, how **often** did you feel sexually aroused ("turned on") during sexual activity or intercourse?	◯ 0 = No sexual activity<br>◯ 1 = Almost always or always<br>◯ 2 = Most times (more than half the time)<br>◯ 3 = Sometimes (about half the time)<br>◯ 4 = A few times (less than half the time)<br>◯ 5 = Almost never or never
**Q4**: Over the past 4 weeks, how would you rate your **level** of sexual arousal ("turn on") during sexual activity or intercourse?	◯ 0 = No sexual activity<br>◯ 1 = Very high<br>◯ 2 = High<br>◯ 3 = Moderate<br>◯ 4 = Low<br>◯ 5 = Very low or none at all

Q5: Over the past 4 weeks, how **confident** were you about becoming sexually aroused during sexual activity or intercourse?

- 0 = No sexual activity
- 1 = Very high confidence
- 2 = High confidence
- 3 = Moderate confidence
- 4 = Low confidence
- 5 = Very low or no confidence

Q6: Over the past 4 weeks, how **often** have you been satisfied with your arousal (excitement) during sexual activity or intercourse?

- 0 = No sexual activity
- 1 = Almost always or always
- 2 = Most times (more than half the time)
- 3 = Sometimes (about half the time)
- 4 = A few times (less than half the time)
- 5 = Almost never or never

Q7: Over the past 4 weeks, how **often** did you become lubricated ("wet") during sexual activity or intercourse?

- 0 = No sexual activity
- 1 = Almost always or always
- 2 = Most times (more than half the time)
- 3 = Sometimes (about half the time)
- 4 = A few times (less than half the time)
- 5 = Almost never or never

Q8: Over the past 4 weeks, how **difficult** was it to become lubricated ("wet") during sexual activity or intercourse?

- 0 = No sexual activity
- 1 = Extremely difficult or impossible
- 2 = Very difficult
- 3 = Difficult
- 4 = Slightly difficult
- 5 = Not difficult

Q9: Over the past 4 weeks, how often did you **maintain** your lubrication ("wetness") until completion of sexual activity or intercourse?

- 0 = No sexual activity
- 1= Almost always or always
- 2 = Most times (more than half the time)
- 3 = Sometimes (about half the time)
- 4 = A few times (less than half the time)
- 5 = Almost never or never

Q10: Over the past 4 weeks, how **difficult** was it to maintain your lubrication ("wetness") until completion of sexual activity or intercourse?

- 0 = No sexual activity
- 1 = Extremely difficult or impossible
- 2 = Very difficult
- 3 = Difficult
- 4 = Slightly difficult
- 5 = Not difficult

Q11: Over the past 4 weeks, when you had sexual stimulation or intercourse, how **often** did you reach orgasm (climax)?

- 0 = No sexual activity
- 1 = Almost always or always
- 2 = Most times
- 3 = Sometimes (about half the time)
- 4 = A few times (less than half the time)
- 5 = Almost never or never

Q12: Over the past 4 weeks, when you had sexual stimulation or intercourse, how **difficult** was it for you to reach orgasm (climax)?

- 0 = No sexual activity
- 1 = Extremely difficult or impossible
- 2 = Very difficult
- 3 = Difficult
- 4 = Slightly difficult
- 5 = Not difficult

Q13: Over the past 4 weeks, how **satisfied** were you with your ability to reach orgasm (climax) during sexual activity or intercourse?

- 0 = No sexual activity
- 1 = Very satisfied
- 2 = Moderately satisfied
- 3 = About equally satisfied and dissatisfied
- 4 = Moderately dissatisfied
- 5 = Very dissatisfied

Q14: Over the past 4 weeks, how **satisfied** have you been with the amount of emotional closeness during sexual activity between you and your partner?

- 0 = No sexual activity
- 1 = Very satisfied
- 2 = Moderately satisfied
- 3 = About equally satisfied and dissatisfied
- 4 = Moderately dissatisfied
- 5 = Very dissatisfied

Q15: Over the past 4 weeks, how **satisfied** have you been with your sexual relationship with your partner?

- 1= Very satisfied
- 2 = Moderately satisfied
- 3 = About equally satisfied and dissatisfied
- 4 = Moderately dissatisfied
- 5 = Very dissatisfied

Q16: Over the past 4 weeks, how **satisfied** have you been with your overall sexual life?

- 1 = Very satisfied
- 2= Moderately satisfied
- 3 = About equally satisfied and dissatisfied
- 4 = Moderately dissatisfied
- 5 = Very dissatisfied

Q17: Over the past 4 weeks, how **often** did you experience discomfort or pain **during** vaginal penetration?

- 0 = Did not attempt intercourse
- 1 = Almost always or always
- 2 = Most times (more than half the time)
- 3 = Sometimes (about half the time)
- 4 = A few times (less than half the time)
- 5 = Almost never or never

Q18: Over the past 4 weeks, how **often** did you experience discomfort or pain **following** vaginal penetration?

- 0 = Did not attempt intercourse
- 1 = Almost always or always
- 2 = Most times (more than half the time)
- 3 = Sometimes (about half the time)
- 4 = A few times (less than half the time)
- 5 = Almost never or never

Q19: Over the past 4 weeks, how would you rate your **level** (degree) of discomfort or pain during or following vaginal penetration?

- 0 = Did not attempt intercourse
- 1 = Very high
- 2 = High
- 3 = Moderate
- 4 = Low
- 5 =Very low or none at all

Scoring System

The individual domain scores and full scale score of the FSFI are derived by the computational formula outlined in the table below. Individual domain scores are obtained by adding the scores of the individual items that comprise the domain and multiplying the sum by the domain factor (see below). The full scale score is obtained by adding the six domain scores. It should be noted that within the individual domains, a domain score of zero indicates that no sexual activity was reported during the past month.

Domain	Questions	Score range	Factor	Minimum score	Maximum score
Desire	1, 2	1-5	0.6	1.2	6.0
Arousal	3, 4, 5, 6	0-5	0.3	0	6.0
Lubrication	7, 8, 9, 10	0-5	0.3	0	6.0
Orgasm	11, 12, 13	0-5	0.4	0	6.0
Satisfaction	14, 15, 16	0 (or 1)-5	0.4	0	6.0
Pain	17, 18, 19	0-5	0.4	0	6.0
		Full Scale Score Range		2.0	36.0

*For the complete FSFI questionnaire, instructions, and scoring algorithm, please see www.FSFIquestionnaire.com, or contact Raymond Rosen PhD, (Department of Psychiatry: UMDNJ-Robert Wood Johnson Medical School, 675 Hoes Lane, Piscataway, NJ 08854)

Reprinted from: Rosen R, Brown C, Heiman J, et al. The Female Sexual Function Index (FSFI): a multidimensional self-report instrument for the assessment of female sexual function." *J Sex Marital Ther*. 2000;26:191-208, with permission of Taylor & Francis, Ltd.
